# Immunomodulation Effects of* Schizonepeta tenuifolia* Briq. on the IgE-Induced Allergic Model of RBL-2H3 Cells

**DOI:** 10.1155/2018/6514705

**Published:** 2018-04-22

**Authors:** Yi-Hsuan Lin, Hsing-Yu Chen, Jung-Chun Chiu, Kun-Jei Chen, Hung-Yao Ho, Sien-Hung Yang

**Affiliations:** ^1^Division of Chinese Internal Medicine, Center for Traditional Chinese Medicine, Chang Gung Memorial Hospital, Taoyuan 33378, Taiwan; ^2^School of Traditional Chinese Medicine, College of Medicine, Chang Gung University, Taoyuan 33302, Taiwan; ^3^Graduate Institute of Clinical Medical Sciences, College of Medicine, Chang Gung University, Taoyuan 33302, Taiwan; ^4^Division of Gastroenterology, Department of Pediatrics, Chang Gung Memorial Hospital, Linkou, Taoyuan 33305, Taiwan; ^5^Healthy Aging Research Center, Chang Gung University, Guishan, Taoyuan 33302, Taiwan; ^6^Clinical Phenome Center, Chang Gung Memorial Hospital, Linkou, Guishan, Taoyuan 33305, Taiwan; ^7^Department of Medical Biotechnology and Laboratory Science, College of Medicine, Chang Gung University, Taoyuan 33302, Taiwan; ^8^Chang Gung Immunology Consortium, Chang Gung Memorial Hospital and Chang Gung University, Guishan, Taoyuan 33302, Taiwan

## Abstract

*Schizonepeta tenuifolia *(ST) Briq. is a traditional herbal medicine commonly used to treat allergic skin diseases, where the inflammation process is closely related to symptom severity. This study aimed to explore the immunomodulatory effect of ST by using immunoglobulin E- (IgE-) stimulated RBL-2H3 cell cultures, a common cell line for studying mast cell degranulation and inflammatory cytokine release* in vitro*. After stimulating the RBL-2H3 cells with IgE, ST at concentrations of 10, 50, or 100 *μ*g/mL was added to the cell cultures. Cell viability, inflammatory cytokines (IL-6, IL-13, IL-4, TNF-*α*, and IFN-*γ*), anti-inflammatory cytokine IL-10, and degranulation ability were examined 48 and 72 hours after administration of ST. The markers of inflammation and allergic reaction, IFN-*γ*, TNF-*α*, IL-4, and IL-6, were suppressed, especially after treatment with 100 *μ*g/mL ST. However, the anti-inflammation marker IL-10 was also suppressed by ST. Trend analysis showed that a higher ST concentration was associated with lower IFN-*γ* and TNF-*α* levels. Moreover, degranulation of RBL-2H3 cells was assessed by measuring the release of *β*-hexosaminidase, which was suppressed by ST at 10 *μ*g/mL. This study showed an immunomodulatory effect of ST at the cellular level and suggests the role of ST in treating allergic diseases.

## 1. Introduction

Mast cells are widely distributed at the mucosal interface and thus play an important role in allergic diseases, such as urticaria, atopic dermatitis, allergic rhinitis, and asthma. Mast cells can be activated by IgE through the high-affinity IgE receptor (Fc*ε*RI) and produce diverse mediators that can promote or downregulate inflammation [1, 2]. Mobilization of Ca^2+^ in response to IgE receptor-mediated signaling is a key process in mast cell activation [3]. Allergic skin diseases, such as urticaria and atopic dermatitis, are highly related to the degranulation of mast cells and the balance between inflammatory and anti-inflammatory cytokines, such as interleukin- (IL-) 4, IL-6, IL-10, IL-13, interferon gamma (IFN-*γ*), and tumor necrosis factor alpha (TNF-*α*) [4, 5]. Therefore, to study the pharmacologic effects of allergic skin diseases, an inducible mast cell line is needed.

Currently, three mast cell lines (HMC-1, LAD2, and LUVA cells) and one basophil cell line (RBL-2H3 cells) are commonly used to study allergic reactions, and here RBL-2H3 cells were chosen to examine the anti-inflammatory effects of* Schizonepeta tenuifolia*, a herb well known for the treatment of allergic skin diseases [6]. HMC-1 cells, which are like immature mast cells, can only express small amounts of tryptase and chymase, with nearly no functional Fc*ε*RI on the cell surface [7, 8]. LAD2 cells are not suitable for these studies, as they have an unstable phenotype and cell doubling time [9]. LUVA cells, which are derived from patients without mast cell-associated diseases, have the advantage of growth in cell cultures without requiring additional growth factors. However, studies on allergic reactions using LUVA cells are still limited. Although derived from basophilic leukemia cells in neonatal Wistar rats, RBL-2H3 cells share similar characteristics to mast cells, and therefore RBL-2H3 cells have been extensively used as a good model for mast cell IgE-mediated degranulation [10–13].

*Schizonepeta tenuifolia* (ST) Briq., which belongs to the family Lamiaceae, is an herbal medicine that has been widely used for thousands of years in China, Taiwan, Japan, and Korea. The dried aerial part of ST is applied clinically for diseases such as allergic skin disease, inflammatory skin disease, infectious skin disease, and the common cold [14, 15]. For this reason, we hypothesize that ST has anti-inflammation and antidegranulation effects on mast cells. Choi et al. stated that ST inhibited 2,4-dinitrochlorobenzene- (DNCB-) induced atopic dermatitis in mice by the suppression of IgE, TNF-*α*, and IL-6 and by nuclear factor kappa-light-chain-enhancer of activated B cells (NF-*κ*B) activation, as well as through mitogen-activated protein kinase (MAPK) activity [16]. Wang et al. also presented the antioxidant and anti-inflammatory role of aqueous extracts of ST in the carrageenan-induced inflammatory response in mice, and the volatile oil from ST also can relieve carrageenan-induced pleurisy in rat [17, 18]. ST also regulates cytokine-cytokine receptor interaction, MAPK, and Toll-like receptor (TLR) signaling pathways in HMC-1 mast cells [19]. Methanol extracts of ST inhibit the substance p-induced itch-scratch response in mice [20]. Furthermore, aqueous extracts of ST inhibit mast cell-mediated immediate-type hypersensitivity, suggesting that ST has the potential to treat allergic diseases including urticaria, angioedema, allergic asthma, and allergic rhinitis [21]. To prove the hypothesis about the immunomodulatory abilities of ST, a water extract of ST was used to suppress inflammation and degranulation of mast cells using the RBL-2H3 cell line in vitro in this study.

## 2. Materials and Methods

### 2.1. Preparation of ST Water Extract

A sample of ST was obtained from a pharmaceutical factory with good manufacturing practices, Chuang Song Zong Co., Ltd. (Kaohsiung, Taiwan). ST was extracted using water (weight 5.2 g, water content 50.47%) and filtered through a 0.22 *μ*M syringe filter. The extract was stored at 4°C and diluted to the required concentrations on the day of use.

### 2.2. RBL-2H3 Cell Culture

RBL-2H3 cells were obtained from the Bioresource Collection and Research Center (Hsinchu, Taiwan). RBL-2H3 cells were cultured in DMEM supplemented with 15% (v/v) heat-inactivated FBS, 2% glutamine, and 100 U/mL penicillin. Cells were grown at 37°C in an atmosphere of 5% CO2 and 95% air. The cells were detached from T75 flasks using a trypsin-EDTA solution and resuspended in fresh medium for subsequent experiments.

### 2.3. Cell Viability Assay (MTT Assay)

Cell viability was determined using a 3-[4,5-dimethylthiazol-2-yl]-2,5-diphenyltetrazolium bromide (MTT) colorimetric assay (Sigma-Aldrich). RBL-2H3 cells were grown in 96-well plates (5 × 105 cells/mL, each well containing 4 × 104 cells). After treating RBL-2H3 cells with different concentrations of ST for 48 hrs, cells were treated with 20 *μ*L MTT (5 mg/mL) and then incubated at 37°C for 2 hrs. Cells were then washed, and the insoluble formazan products were dissolved in 150 *μ*L 0.1% Triton X-100 acidified isopropanol (13 *μ*L 37% HCl, 40 *μ*L Triton X-100, and 40 mL isopropanol). After shaking for 15 min, absorbance was measured at the 570 nm wavelength by spectrophotometry (Bio-Tek Instruments, Winooski, VT, USA). All measurements were made in triplicate, and a total of three independent experiments were performed. Cell viability data were expressed as the percentage relative to the viability in control cell cultures.

### 2.4. Determination of Cytokine Levels

The inhibitory effects of ST at concentrations of 10, 50, or 100 *μ*g/mL on the IgE-stimulated release of cytokines in RBL-2H3 cells were evaluated. For this, RBL-2H3 cells were activated with antidinitrophenyl (DNP) IgE (0.5 *μ*g/ml; Sigma-Aldrich) overnight, and then the three different concentrations of ST were added for 48 hrs and 72 hrs, separately. After treatment, cells were challenged with DNP-bovine serum albumin (BSA; 100 ng/ml; Sigma-Aldrich) for 45 min at 37°C and then incubated on ice for 10 min. Culture supernatants from RBL-2H3 cells were assayed in triplicate for IL-4, IL-6, IL-10, IL-13, IFN-*γ*, and TNF-*α* using ELISA kits according to the manufacturer's instructions (eBioscience, San Diego, CA, USA).

### 2.5. Analysis of *β*-Hexosaminidase Release

To measure *β*-hexosaminidase release, RBL-2H3 cells were trypsinized and centrifuged, before resuspension in DMEM-FCS and stimulated with monoclonal anti-DNP IgE (0.5 *μ*g/ml; Sigma-Aldrich) overnight. The cells were washed with Siraganian buffer (119 mM NaCl, 5 mM KCl, 5.6 mM glucose, 0.4 mM MgCl_2_, 25 mM PIPES, 40 mM NaOH, 1 mM CaCl_2_, and 0.1% BSA) and then treated with or without ST at the indicated concentrations. After incubation for 10 min at 37°C, the cells were challenged with DNP-BSA (100 ng/ml; Sigma-Aldrich) for 45 min at 37°C and then put on ice for 10 min. Culture supernatants were incubated with 1 mM p-nitrophenyl-N-acetyl-ß-D-glucosaminide (p-NAG; Sigma-Aldrich) for one hr at 37°C and then 0.1 N Na2CO3/NaHCO3 (pH 10.0) was added to stop the reaction. Optical density at 405 nm was measured. The inhibition of *β*-hexosaminidase granule release was calculated using the following equations:

(1) $$\begin{array}{c} \%\text{ of inhibition}=\frac{\left(\text{Treated}-\text{Blank}-\text{Spontaneous}\right)}{\left(Control-Blank-Spontaneous\right)}, \end{array}$$

where the Control was defined as the normal allergen-IgE response evoked without ST added; Treated was defined as the normal allergen-IgE response evoked with added ST; Blank was only ST and substrate added to the ELISA plate; and Spontaneous meant that the allergen-IgE response was not evoked and ST was not added.

### 2.6. Statistical Methods

All data and statistic tests were processed using SPSS (version 15.0, Chicago, IL, USA) and represented as mean ± standard error of mean (SEM). The differences between groups were analyzed using the nonparametric Wilcoxon signed-rank test. The trend analyses for dose-dependent relations between ST concentration and inflammatory cytokines were performed using one-way analysis of variance (ANOVA). A *p* value less than 0.05 was considered statistically significant.

## 3. Results

### 3.1. ST Has No Significant Influence on RBL-2H3 Cell Viability

**Figure 1:**
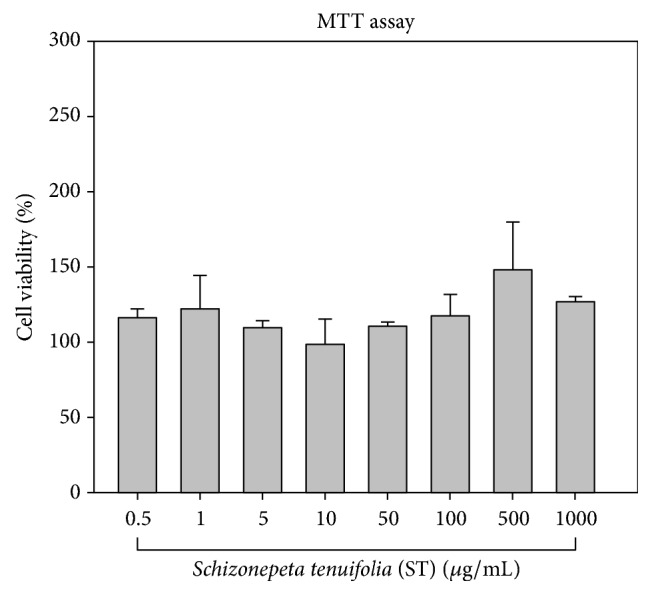
*Schizonepeta tenuifolia* (ST) Briq. has no significant negative effects on RBL-2H3 cells. MTT was used to assess cell viability after treatment with different concentrations of ST. When compared to the control, set as 100%, no cell toxicity was noted after treatment with 10, 50, and 100 *μ*g/mL ST.

First, we confirmed there was no cytotoxicity following treatment with ST at any concentration. Even at high concentrations of ST treatment (1000 *μ*g/mL), cell toxicity was not observed (Figure 1). We used ST at concentrations of 10, 50, and 100 *μ*g/mL in the following experiments.

### 3.2. Effects of ST on Proinflammatory Cytokine Production in IgE-Stimulated RBL-2H3 Cells

**Figure 2:**
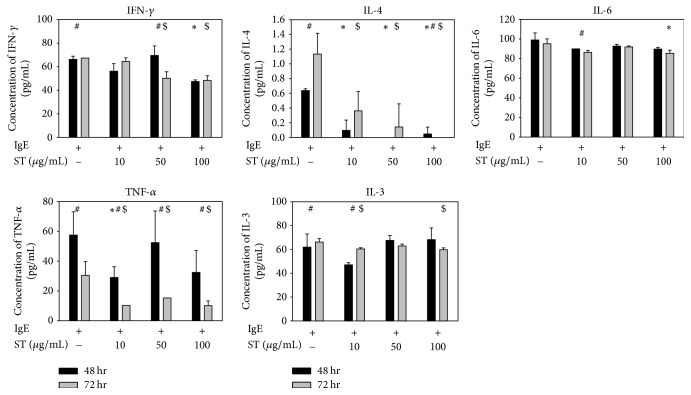
The evolution of proinflammatory cytokines 48 and 72 hrs after treating IgE-induced RBL-2H3 cells with 10, 50, or 100 *μ*g/mL ST shows that the inflammatory cytokines released by RBL-2H3 cells tend to decrease with increasing doses of ST, especially IFN-*γ* and TNF-*α*. ^*∗*^*p* < 0.05 when compared with controls after 48 hrs of ST treatment; ^$^*p* < 0.05 when compared with controls after 72 hrs of ST treatment; and ^#^*p* < 0.05 when comparing data between 48 and 72 hrs of treatment.

As showed in Figure 2, IFN-*γ*, IL-4, IL-6, TNF-*α*, and IL-13 significantly decreased in the ST group when compared to the control group. IL-4 in IgE-stimulated RBL-2H3 cells decreased after treatment with ST for 48 and 72 hrs, which were both statistically significant compared to controls. The decreases in IL-4 and TNF-*α* were most prominent, especially for IL-4, as the concentration of IL-4 barely shown in the 100 *μ*g/mL ST treatment group. In addition, the trend analysis showed that higher concentrations of ST may relate to lower levels of IFN-*γ* and TNF-*α*.

### 3.3. Effects of ST on IL-10, an Anti-Inflammatory Cytokine in IgE-Stimulated RBL-2H3 Cells

**Figure 3:**
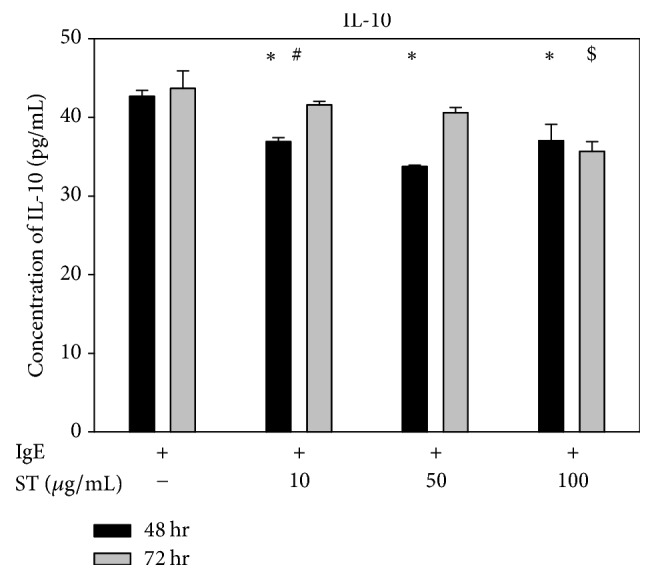
The anti-inflammatory cytokine IL-10 did not increase in RBL-2H3 cells subjected to IgE and ST. Bars show the evolution of IL-10 48 and 72 hrs after treating IgE-induced RBL-2H3 cells with ST at 10, 50, or 100 *μ*g/mL. ^*∗*^*p* < 0.05 when compared with controls after 48 hrs of ST treatment; ^$^*p* < 0.05 when compared with controls after 72 hrs of ST treatment; and ^#^*p* < 0.05 when comparing data between 48 and 72 hrs of treatment.

Figure 3 showed that IL-10, an anti-inflammatory marker in IgE-induced RBL-2H3 cells, decreased after treatment with ST. The concentration of IL-10 lowered as ST concentration increased (trend analysis, *p* < 0.05), and the concentration of IL-10 was generally lower at 48 hrs but increased at 72 hrs, except in cell cultures treated with 100 *μ*g/mL ST, although the difference between 48 and 72 hours of cell culture did not reach significance.

### 3.4. Effects of ST on *β*-Hexosaminidase Release

**Figure 4:**
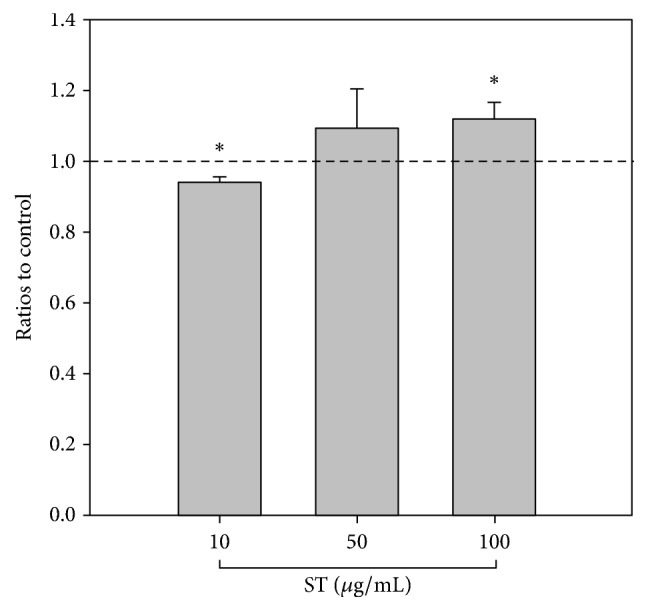
*β*-Hexosaminidase release decreased in IgE-stimulated RBL-2H3 cells subjected to the ST treatment (10 *μ*g/mL). However, the release of *β*-hexosaminidase seemed higher after treatment with 50 and 100 *μ*g/mL ST. The concentration of *β*-hexosaminidase was set to 1 in the control group (dashed line). ^*∗*^*p* < 0.05 when compared to controls.

Furthermore, the release of *β*-hexosaminidase was examined to assess the cytokine secretory ability of IgE-stimulated RBL-2H3 cells. In comparison with the control group, the *β*-hexosaminidase level was significantly lower in the group treated with 10 *μ*g/mL ST, but the inhibition of stimulated cells was not observed in the groups treated with 50 or 100 *μ*g/mL ST (Figure 4). The trend analysis showed no dose-dependent relationships between different doses of ST and *β*-hexosaminidase release.

## 4. Discussion

In this study, we demonstrated that ST may have anti-inflammatory effects on IgE-stimulated RBL-2H3 cells, a model for allergic disease, and the effect may not be related to degranulation ability. Levels of IL-4, IL-6, IFN-*γ*, and TNF-*α* were all much lower in the ST-treated groups than in the control group, and the decreasing trend seemed roughly both dose- and duration-dependent. In addition, the level of IL-13 was lower among the ST treatment group (at 10 *μ*g/mL), whereby IL-13 is thought to be a marker of urticaria severity [22]. IL-4 was the first cytokine found to be produced by mast cells and is responsible for IL-13 production in mast cells [23]. During the allergic response, mast cells produce IL-4 rapidly to stimulate an inflammatory response [24]. Then, IL-4 raises the production of IL-13 in these cells [25]. IL-6 is an important cytokine for mast cell maturation and the upregulation of histamine production [26]. TNF-*α* is a potent mast cell chemoattractant, which may promote inflammation among mast cells and subsequent dendritic cell migration [27–29]. In addition to mast cells, ST was reported to suppress both Th1 and Th2 cells, the balance of which is regarded as an important treatment target [30, 31]. The multicell and extensive immunomodulation effect of ST may be useful for skin diseases, especially when allergy and inflammation are both concerned. IL-10 can serve as a natural regulator of mast cell homeostasis by dampening mast cell Fc*ε*RI expression, preventing excessive activation and inflammation [32]. Since IL-10 was not lower in the ST treatment groups, this may imply that ST suppresses both the inflammatory and anti-inflammatory response at the same time. Since mast cells may have both immunostimulatory and immunosuppressive actions, the dual suppression effect may be needed for mast cell related skin diseases [33].

Moreover, the immunomodulation effect of ST seems less relevant to the inhibition of mast cell degranulation, which may have two explanations: certain inflammatory cytokines, such as TNF and IL-6, were not released from mast cells by degranulation, or ST suppressed the release of inflammatory cytokines through other pathways. Allergic symptoms are caused by both unbalanced cytokines and the abnormal degranulation of mast cells. The activation of mast cells contributes to the pathophysiology of many allergic diseases through the synthesis and release of numerous proinflammatory mediators and cytokines. Mast cells can express different receptors and ligands on the cell surface, and after activation they release mediators such as histamines, leukotrienes, and cytokines chemokines; all of these mediators and cell surface molecules can promote inflammation in the skin [34]. Mast cells may also be able to selectively release proinflammatory mediators without the degranulation typical of allergic reactions; for example, IL-1 may induce the selective release of IL-6 [33, 35]. Also, TNF and IFN-*γ* could be released without evidence of degranulation [33]. In contrast, ST may suppress inflammation via the activation of toll-like receptor 4 (TLR4), which may further decrease the release of TNF-*α* while leaving degranulation unaffected [35, 36].

Nevertheless, the unaffected degranulation and suppressed secretion of IL-10 may be the reason that ST is commonly used with other herbs when treating allergic skin diseases; for example, ST is a part of a complicated herbal network for the treatment of urticaria [6, 37]. ST is the major ingredient in the most commonly used herbal prescription for urticaria and the twelve other herbs used may complement the treatment effect by suppressing histamine release and increasing the anti-inflammation effects [38]. A more extensive effect on urticaria can thus be found when these herbs are used in combination.

Finally, we found RBL-2H3 cells to be a feasible in vitro model to examine the mechanisms and effects of compounds on urticaria. In our study, the anti-inflammatory response in RBL-2H3 cells was like findings describing the response in the mast cell model, HMC-1 cells, as both systems showed the IL-6 and TNF-*α* suppression [19, 21]. Although basophils and mast cells have different cellular origins, both cells are important in chronic urticaria for the release of histamine when exposed to the susceptible allergens [39, 40]. Moreover, basophils share many similarities with mast cells, such as similar cell morphology under staining, intracellular granule contents, and the secretory route of cytokines [10]. These features make RBL-2H3 cells a good candidate cell line to examine the activity of urticaria at the cellular level and could also be used as a drug screening platform. However, we found that some secretory cytokines may have relatively large variations under specific conditions, such as the TNF-*α* level at 48 hrs and the IL-4 level at 72 hrs after stimulated cells were subjected to ST, which has also been seen in other experiments using RBL-2H3 cells [41]. Likewise, a prior report also points out different behaviors between RBL-2H3 cells and mast cells [41]. For this reason, studies evaluating the overall effects of compounds on urticarial should be based on more extensive inflammatory/anti-inflammatory cytokines profiles.

## 5. Conclusions

Mast cells are important sources of cytokines in allergic and inflammatory diseases. Targeting mast cell-derived cytokines could be a beneficial treatment strategy for allergic inflammatory diseases. Currently, broadly immunosuppressive drugs, such as corticosteroids, are used to treat allergic inflammation. Discovering a therapy for allergic inflammatory diseases with fewer side effects was an aim of this study. Based on the existing data, ST may be a possible treatment for allergic inflammatory diseases via targeting anti-inflammatory pathways. ST, a natural Chinese herbal medicine, could bring hope to patients with chronic allergic inflammatory diseases. With further research, additional inflammatory allergic diseases, such as urticaria, atopic dermatitis, asthma, and allergic rhinitis, may benefit from ST treatment. Based on our results, further animal studies and human trials will be necessary to complete this aim. The authors hope that successful experiments and trials could introduce ST as a novel treatment for the allergic inflammatory disease to improve patients' quality of life.
